# Conflict-related violence and mental health among self-settled Democratic Republic of Congo female refugees in Kampala, Uganda – a respondent driven sampling survey

**DOI:** 10.1186/s13031-021-00377-2

**Published:** 2021-05-26

**Authors:** Itziar Familiar, Pamela Nasirumbi Muniina, Chris Dolan, Moses Ogwal, David Serwadda, Herbert Kiyingi, Chantal Siya Bahinduka, Enos Sande, Wolfgang Hladik

**Affiliations:** 1 Department of Psychiatry, Michigan State University, East Lansing, MI USA; 2 Division of Global HIV and TB, Centers for Disease Control and Prevention, Kampala, Uganda; 3 Refugee Law Project, School of Law, Makerere University, Kampala, Uganda; 4 School of Public Health, Makerere University, Kampala, Uganda; 5 Department of Disease Control, School of Public Health, Makerere University, Kampala, Uganda; 6 Action Marguerite, Winnipeg, Manitoba Canada; 7 Division of Global HIV and TB, Centers for Disease Control and Prevention, MS E-30, 1600 Clifton Rd, Atlanta, GA-30333 USA

**Keywords:** DR Congo, Uganda, Refugees, Female, Rape, Mental health

## Abstract

**Background:**

Violence and traumatic events are highly prevalent among refugees, but less is known about the impact of these experiences among self-settled refugees in the country of asylum. We evaluated the association between traumatic experiences and PTSD and depression symptoms among female Democratic Republic of Congo (DRC) refugees living in Kampala, Uganda.

**Methods:**

Participants were recruited using respondent driven sampling in one refugee service center in Kampala, Uganda. Eligibility criteria included: Congolese nationality, age 18+ years, self-settled in Kampala for at least 6 months, refugee status or documentation of application for refugee status. Only data from female participants were included in this analysis. Depression symptoms were screened with the Patient Health Questionnaire-2, and symptom criteria for PTSD and traumatic experiences were evaluated with the Harvard Trauma Questionnaire. Logistic regression models were performed to separately assess associations between mental health outcomes (PTSD and depression), rape and non-sexual violence.

**Results:**

Five hundred eighty women with a mean age of 33 years were interviewed. Among participants, 73% (95% CI:67–78%) met symptom criteria for PTSD, 57% (95% CI: 51–63%) for depression, and 65% reported thoughts of ending one’s life. 79% of women reported experience of rape, for over half (54%) it occurred more than once, and 82% were gang raped. Crude and adjusted odds ratios (ORs) show that PTSD was most strongly associated with being raped (OR = 2.43, *p* < 0.01), lacking shelter (OR = 2.86, *p* < 0.01), lacking food or water (OR = 2.53, *p* = 0.02), lacking access to health care (OR = 2.84, *p* < 0.01), forced labor (OR = 2.6, *p* < 0.01), extortion and/or robbery (OR = 3.08, *p* < 0.01), experiencing the disappearance/kidnapping of a family member or friend (OR = 2.72, *p* < 0.01), and witnessing the killing or murder of other people (OR = 3.28, *p* < 0.01). Depression was significantly associated with several traumatic experiences including rape (OR = 2.3, *p* = 0.01), and experiencing the disappearance/kidnapping of a child or spouse (OR = 1.99, *p* = 0.01).

**Conclusions:**

Refugee women self-settled in Kampala reported high lifetime experiences of violence and traumatic events including rape, as well as high rates of PTSD and depression. Future programming addressing self-settled refugees and their settlement in host countries may benefit from including local and national integration strategies.

## Background

Since the first and second Congo Wars in 1996–1997 and 1998–2003, respectively, political instability and armed conflict has forced an estimated 4.5 million people to flee from the Democratic Republic of Congo (DRC). The UN High Commissioner for Refugees [1] estimated that as of February 2019, over 826,000 DRC refugees were being hosted in African countries, with as many as 60% of them currently living in Uganda [2]. The multiple armed conflicts affecting DRC’s vast territory make this one of the most challenging humanitarian emergencies of our time. A tragic feature of the armed conflict in the DRC has been the use of widespread sexual violence against women, including rape, as a tactic of war and destabilization [3].

The events leading to forced migration, as well as flight itself and life in the country of asylum, are frequently accompanied by exposure to an array of traumatic events such as severe physical and sexual violence, death threats, witnessing family and others being killed, kidnapped, or abused [4]. These events can be emotionally shocking, and rates of stress and mental health disorders, such as anxiety disorders, post-traumatic stress disorder (PTSD), and depression are higher among refugees than in the general population [5].

Women are more frequently victims of sexual violence and the prevalence of PTSD and poor mental health after rape is particularly high. A systematic review and meta-analysis estimated the prevalence of sexual violence and rape among female refugees and those internally displaced at 21% (CI 95% 14.9–28.7) [6], with authors warning about the potential underestimation due to barriers in reporting. In the DRC, population-based studies show that more than 40% of women have experienced sexual violence including rape [7, 8] and upwards of 50% among female refugees [9]. Studies of DR Congolese civilians and refugees have found higher prevalence of PTSD, depression, suicidal ideation, and suicidal attempts among women who have experienced sexual violence [7, 10].

Most studies describing mental health issues of refugees are based on reports from individuals assessed within organized temporary settlements. Less is known about the considerable large number of refugees who have chosen to ‘self-settle’ within a host population. Self-settled and urban refugees may be more vulnerable to exploitation, persecution and arrest, given that although they are recognized as refugees, there is little in the way of practical assistance or protection to those living outside gazetted refugee settlements [2]. Ensuring support and access to basic services for individuals concealed in the anonymity of a city can be challenging, increasing the likelihood of risk-taking behaviors, and negative health outcomes [1]. Self-settled refugees may experience higher levels of violence and rape with even less support available than what refugees in camps may have access to from medical non-governmental organizations (NGOs). Determining the type and magnitude of mental health problems urban refugees experience is crucial to plan and deliver adequate services, and requires methods that can be used to generate valid estimates for hidden populations that do not have a sampling frame.

The purpose of this study was to investigate rates of PTSD and depression symptoms in a population of female DR Congolese refugees living in Kampala, Uganda and their association with traumatic experiences including rape, to inform policy and service provision in host countries.

## Methods

Data come from the Crane Survey, a cross-sectional survey conducted in Kampala, Uganda between July 25th and November 29th, 2013 by the Crane Survey of Makerere University School of Public Health, the US Centers for Disease Control and Prevention, and the Refugee Law Project.

### Participant recruitment

Study participants were recruited using respondent-driven sampling (RDS), a peer-to-peer recruitment approach used to sample hard-to-reach populations where no sampling frame is available. A single survey office located in a dense urban area near the city center was used as the survey site. Survey participation involved two visits to the survey office. In the first visit, the coupon received by one of two seeds was validated, and candidate participants were screened in a face-to-face interview for eligibility (DR Congolese nationality, age 18+ years, self-settled in Kampala for at least 6 months, self-reported refugee status or documentation of application for refugee status). If eligible, and after providing informed consent, participants were interviewed and offered testing for syphilis and HIV. A second visit was scheduled 2 weeks later, to return test results, administer a short peer recruitment interview and provide compensation for transport and participation as detailed below.

### Procedure

#### Instruments

All instruments were translated into Swahili and French and back translated by local research assistants. To increase confidentiality and data protection, survey instruments were administered to participants using an audio computer-assisted self-interview (ACASI) in a password protected tablet using Questionnaire Design Studio (Nova Research). Research staff introduced the ACASI format to participants through a tutorial and then left the participant to provide privacy but remained within sight or earshot to troubleshoot as needed. ACASI accommodates low and illiterate respondents. The few participants who were overwhelmed by the computer setting were offered a computer-assisted personal interview. Data were entered and merged in a password protected study laptop only accessed by authorized study personnel and de-identified before analyses.

To estimate the personal network size, participants were asked “How many [self-settled DR Congolese refugees] do you know by sight or name?”, continuing with the following probes: “Of these, how many live in Kampala?”, “How many are aged 18 or older”, and “Of these, how many could you give a survey coupon by this time next week?”. Throughout the survey procedures, Congolese survey volunteers helped with communication and explanations as needed, in addition to the main Ugandan survey staff.

Mental health outcomes were assessed using questionnaires previously administered in refugee settings. The validated short form of the Patient Health Questionnaire-2 (PHQ-2) [11] was used to screen for depressive symptomatology. The PHQ-2 inquires on the frequency of anhedonia and depressed mood in the past 2 weeks. Scores range from 0 to 6 and the established cut-off value of 3 was used to denote probable depression. Finally, one binary (yes/no) question asked about thoughts of ending one’s own life.

PTSD was assessed with the first 16 trauma symptom items of the Harvard Trauma Questionnaire (HTQ), derived from the DSM-IV PTSD criteria [12]. Respondents endorsed how much each symptom bothered them in the past week using a four-point Likert scale (not at all, several days, more than half the days, nearly every day). The HTQ-PTSD score is an average score, with a cut-off score of 2.5 used to suggest that the respondent has a high likelihood of clinically meaningful PTSD. Cronbach’s alpha for the HTQ-PTSD was 0.85. Part I of the HTQ (46 item version) was used and adapted to inquire about traumatic events experienced before fleeing, including physical and sexual violence. Rape was defined answering yes to the HTQ item (“Have you experienced rape”) and follow up questions to positive responses were added to collect additional data including, gang rape (defined as two or more persons involved in the rape incident), number of incidents, time of rape (before, during or after displacement), location, perpetrator, injuries sustained, report to authorities, and services received. Social support was measured through the Multidimensional Scale of Perceived Social Support (MSPSS) [13], a brief research tool designed to measure perceptions of social support from three sources; family, friends and a significant other. The scale is comprised of 12 items answered with a 7-point Likert scale, which in this survey were collapsed into a five-point Likert scale (1 = strongly disagree, 2 = mildly disagree, 3 = neutral, 4 = mildly agree, 5 = strongly agree) to accommodate the ACASI formatting (‘very strongly disagree’ and ‘very strongly agree were’ eliminated). Following standard scoring and cut-off guidance [13], a total mean score was calculated, with any mean total scale score ranging from 1 to 2.9 considered low support, a score of 3–5 considered moderate support, and a score > 5.1 considered as high support. Cronbach alpha for the MSPSS was 0.9.

### Ethical considerations

All participants provided oral informed consent for their participation in the survey and testing. No personal identifiers were recorded. Participants who tested positive for syphilis were offered treatment at the survey site according to the Ugandan Ministry and World Health Organization guidelines. Participants who tested HIV-positive received a referral letter for a provider of their choice in Kampala. Self-administered interview responses in ACASI were not reviewed in real time. However, high response values (i.e. Likert response of 4 or “Extreme”) to specific HTQ items were flagged (recurrent thoughts or memories of most terrifying events, recurring nightmares, unable to feel emotions), as well as “thoughts of harming oneself”, and alerted study staff to talk with the participant and offer referrals to counseling services at Refugee Law Project. Participants who expressed suicidal ideation during interview, or at any other survey stage were counseled and given a mental health-related referral to Butabika National Referral Hospital. Compensation for participants’ time, transport and peer-referral efforts amounted to $5.2 USD for their first survey visit and $5.4 USD for the return visit (for an average of successfully referring 1.5 recruits). The survey protocol was approved by the ethics committee of Makerere University’s School of Public Health and the Uganda National Council of Science and Technology and was reviewed by CDC’s Associate Director of Science. This project was also reviewed in accordance with the Centers for Disease Control and Prevention (CDC) human research protection procedures and was determined to be research, but CDC investigators did not interact with human subjects or have access to identifiable data or specimens for research purposes.

### Statistical analysis

Analysis of recruitment patterns and potential biases was done with RDS-Analyst [14]. Estimates were adjusted to account for the non-random sampling using RDS-Analyst population proportion weighting generated from the mean network size. All further data analysis was done using STATA 11 [15]. Key demographics, violence, and mental health outcomes were summarized and reported as mean + − SD or proportion. Unadjusted and adjusted regression models were performed to separately assess associations between mental health outcomes (a continuous PTSD score and dichotomous depression variable) and sexual (e.g. rape) and non-sexual violence reported. Adjusted analyses included variables with a significant association (i.e. *p* < 0.05) in bivariate analyses with outcomes (age, educational status, marital status, and level of social support) (results not shown). Odds ratios (OR) were used to estimate risk and 95% CI for crude and multivariate models. All tests were two-sided and *p* < 0.05 was considered significant.

## Results

Research assistants in conversation with investigators purposefully selected and recruited two seeds who self-reported large personal network sizes. Seed participants were initially given three coupons per recruit. A total of 1560 participants were initially assessed, of which 223 did not meet the full inclusion criteria and were not included. Total coupon uptake was 0.76 (1560 redeemed/ 2051 issued). In total, 1337 participants were eligible and completed the first visit, of which 580 were women and were included in this analysis.

Women were on average 33.7 years of age, 42% had completed between 8 and 12 years of schooling, and the majority (66%) were from the North Kivu province in DRC. Seventy-eight percent had been residing in Uganda less than 6 years and 23% had been in a refugee camp before settling in Kampala. At the time of the survey, women reported being mostly married (42%), unemployed (43%), and living with family (79%) (Table 1).

**Table 1:** Demographic characteristics of Congolese women living in Kampala, Uganda

Characteristic	Value (***n*** = 580)	Weighted percentage (95% CI)
Age		
18–24	121	20.3 (16.1–25.3)
25–34	210	37.1 (31.5–43.1)
35+	249	42.6 (36.7–48.8)
Mean age		33.7 (32.3–35.0)
Residence in DRC (Province)		
North Kivu	354	65.8 (59.2–71.8)
South Kivu	153	31.2 (25.3–37.7)
Other	17	3.1 (1.4–6.4)
Duration of residence in Uganda		
1–5 years	442	78.1 (73.1–82.4)
6–10 years	115	19.4 (15.3–24.4)
> 10 years	23	2.5 (1.4–4.2)
Mean duration		4.1 (3.8–4.4)
Education (Years in school)		
No school	42	10.2 (6.8–15.0)
Primary (1–7 years)	189	35.9 (30.3–42.0)
Secondary (8–12 years)	269	41.8 (36.1–47.9)
Higher education (13+ years)	80	12.0 (8.6–16.5)
Mean years in school		8.2 (7.6–8.7)
Marital status		
Never married	175	27.2 (22.4–32.6)
Married	222	42.5 (36.6–48.7)
Divorced/Separated	81	12.8 (8.3–19.6)
Widowed	102	17.5 (13.0–23.0)
Lives with family	485	79.5 (73.7–84.3)
Current work		
Manual labor	227	34.0 (28.6–39.9)
Skilled labor	24	5.3 (2.9–9.6)
Study	14	3.0 (1.4–6.3)
Unemployed	227	42.7 (36.7–48.8)
Other	88	15.1 (11.3–19.7)
Currently employed (Yes)	339	54.4 (42.9–69.2)
Lived in a refugee camp before living in Kampala	109	22.8 (17.9–28.4)
Source of refugee assistance/support		
Refugee Law Project	132	20.6 (16.3–25.6)
Interaid	362	59.0 (52.9–64.9)
UNHCR	155	23.8 (19.3–29.1)
At least one	478	80.4 (85.0–74.8)
None	102	19.6 (15.0–25.2)

Among participants, 73% (95% CI: 67–78%) met symptom criteria for PTSD, 57% (95% CI: 51–63%) for depression, and 65% reported thoughts of ending one’s life. Two-thirds of women reported high social support levels (62% for the total score, 66% for family support, 66% for support from friends, and 66% from significant others).

Eighty percent of women reported having been raped and over half (55%) reported more than one rape incident. Of those raped, the majority of women reported being gang raped (82%), with 39% of gang-rape victims reporting more than 4 perpetrators involved at a time. 61% of women described the rape occurring before displacement and 50% identified the police or government soldiers as perpetrators. Consequences of  rape included, 87% reported genital injuries, 18% developed a fistula, and 26% resulted in pregnancies (Table 2). 47% of women declared reporting the rape incident to authorities, and 57% received some type of health care.

**Table 2:** Sexual violence reported by Congolese women living in Kampala, Uganda

Characteristic	Value (*n* = 580)	Weighted percentage (95% CI)
Raped	454	79.5 (74.3–83.9)
No. rape incidents		
Once	210	45.5 (38.8–52.4)
More than once	244	54.5 (47.6–61.2)
Time of rape		
Before displacement	285	61.0 (54.1–67.5)
During displacement	153	35.4 (29.1–42.3)
After displacement	104	20.6 (15.7–26.5)
Place where rape occurred		
DRC	404	89.4 (84.3–93.0)
Uganda	108	19.4 (14.9–24.8)
Other	14	3.4 (1.8–6.4)
Rape perpetrator		
Militia	108	23.5 (18.1–30.0)
Police/ Government solders	247	50.5 (43.6–57.3)
UN/ NGO staff	41	11.1 (7.3–16.5)
Other (neighbor, relative, stranger)	110	23.6 (18.5–29.7)
Gang raped (Yes)	371	81.7 (75.8–86.4)
Gang rape frequency (*N* = 371)		
Once	196	55.0 (47.3–62.4)
More than once	175	45.0 (35.9–55.8)
Mean		2.4 (1.8–3.0)
No. of gang rape perpetrators		
< 4	211	61.4 (53.9–68.5)
4+	160	38.6 (31.5–46.1)
Mean		4.5 (3.7–5.3)
Raped with an object	213	46.0 (39.3–52.8)
Received health care for rape injuries	263	57.5 (50.6–64.1)
Received HIV post exposure prophylaxis	195	45.7 (38.9–52.6)
Reported rape to authorities	226	47.3 (40.5–54.1)
Sustained genital injuries	393	86.7 (81.8–90.4)
Fistula as a result of rape	60	18.3 (13.1–24.9)
Pregnancy as result of rape	132	26.2 (20.7–32.5)

Non-sexual traumatic incidents reported included experiencing a combat situation (93%), lack of food or water (88%), destruction of property (84%), physical abuse (84%), forced separation from family (84%), lack of shelter (83%), lack of access to health care (79%), extortion or robbery (78%), and witnessing beatings/torture (74%) (Table 3).

**Table 3:** Other trauma reported by Congolese women living in Kampala, Uganda

Characteristic	Value (*n* = 580)	Weighted percentage (95% CI)
Lack of shelter	492	82.7 (77.5–86.9)
Lack of food or water	511	87.9 (83.2–91.4)
Lack of access to health care	453	79.2 (74.1–83.5)
Destruction of property	505	84.3 (79.1–88.4)
Experience of combat situation	527	92.7 (89.3–95.1)
Physical abuse	473	84.2 (79.8–87.7)
Knifing	224	37.6 (32.0–43.7)
Torture	356	60.9 (54.8–66.7)
War-related injury	306	54.0 (47.9–60.0)
Imprisonment	106	18.1 (13.9–23.1)
Forced labor	244	43.5 (37.6–49.6)
Extortion	467	77.9 (72.4–82.7)
Kidnapped	314	55.1 (49.0–61.0)
Forced separation from family	492	84.1 (79.5–87.8)
Forced to find and bury bodies	161	27.8 (22.7–33.5)
Prevented from burying someone	163	29.5 (24.3–35.4)
Forced to desecrate bodies or graves	33	6.4 (4.0–10.3)
Forced to physically harm another person	90	18.3 (13.9–23.7)
Forced to betray family or friend	39	10.0 (6.5–15.1)
Experienced violent death of spouse or child	192	32.9 (27.4–38.9)
Experienced other family member or friend’s violent death	228	39.8 (34.0–45.9)
Experienced disappearance, kidnapping of spouse or child	285	48.5 (42.5–54.6)
Experienced disappearance, kidnapping of family or friend	281	42.0 (36.2–48.0)
Witness physical injury of family or friend	319	58.0 (52.0–63.8)
Witnessed beating/torture	424	74.0 (68.2–79.1)
Witnessed killings or murders	294	49.9 (43.8–56.0)
Number of traumatic experiences		
<=10 traumatic experiences	133	21.8 (17.3–27.0)
Between 11 and 15 traumatic experiences	255	43.9 (37.9–50.0)
> 15 traumatic experiences	192	34.3 (28.8–40.4)

Crude and adjusted ORs show that PTSD was most strongly associated with being raped (OR = 2.43, *p* < 0.01), being raped after displacement (OR = 3.17, *p* < 0.001), lacking shelter (OR = 2.86, *p* < 0.01), lacking food or water (OR = 2.53, *p* = 0.02), lacking access to health care (OR = 2.84, *p* < 0.01), forced labor (OR = 2.6, *p* < 0.01) extortion and/or robbery (OR = 3.08, *p* < 0.01), experiencing the disappearance/kidnapping of a family member or friend (OR = 2.72, *p* < 0.01), and witnessing the killing or murder of other people (OR = 3.28, *p* < 0.01) (Table 4). Number of traumatic experiences reported had a dose-response relationship with PTSD; women reporting at least 11 different incidents and above 15 had higher odds of PTSD (OR = 1.21, CI 95% 1.12–1.29, *p* < 0.001; OR = 2.15, CI 95% 1.42–3.24, *p* < 0.001, respectively) than those reporting 10 or fewer.

**Table 4:** Association between PTSD score and sexual and non-sexual traumatic experiences

Type of violence	PTSD				Goodness of fit	
	Unadjusted OR (95% CI)	*p*-value	Adjusted OR (95% CI)^a^	*p*-value	F-statistic	*p*-value
**Sexual violence**						
Rape	2.24 (1.2–4.18)	0.01	2.43 (1.27–4.65)	0.01	0.70	0.71
Gang rape	2.04 (0.97–4.28)	0.06	1.92 (0.91–4.06)	0.09	1.95	0.05
Pregnancy resulting from rape	1.57 (0.77–3.20)	0.22	1.70 (0.85–3.44)	0.13	0.82	0.59
Rape before displacement	0.79 (0.40–1.57)	0.50	0.90 (0.45–1.81)	0.77	2.14	0.02
Rape during flight	0.93 (0.47–1.85)	0.84	0.86 (0.67–0.43)	1.73	0.89	0.53
Rape after displacement	3.06 (1.35–6.92)	< 0.001	3.17 (1.37–7.37)	< 0.001	0.61	0.79
**Non-sexual traumatic experiences**						
Lack of shelter	3.02 (1.52–5.99)	0.00	2.86 (1.43–5.74)	0.00	1.01	0.43
Lack of food/water	2.53 (1.14–5.62)	0.02	2.53 (1.18–5.43)	0.02	0.89	0.53
Lack of access to health care	289 (1.57–5.30)	0.00	2.84 (1.55–5.23)	0.00	0.49	0.88
Destruction of property	2.38 (1.13–4.98)	0.02	2.48 (1.18–5.22)	0.02	0.47	0.90
Sustained beatings	1.91 (1.03–3.53)	0.04	1.87 (1.01–3.49)	0.05	1.53	0.13
Tortured	1.61 (0.92–2.82)	0.10	1.71 (0.97–3.03)	0.06	0.74	0.67
War-related injury	2.14 (1.22–3.73)	0.01	2.04 (1.16–3.57)	0.01	0.85	0.57
Imprisonment	1.81 (0.91–3.61)	0.09	1.73 (0.86–3.45)	0.12	3.77	< 0.001
Forced labor	2.77 (1.50–5.12)	0.00	2.60 (1.39–4.85)	0.00	0.48	0.89
Extortion, robbery	3.28 (1.75–6.14)	< 0.001	3.08 (1.61–5.89)	0.00	2.99	0.002
Kidnapped	1.66 (0.96–2.88)	0.07	1.65 (0.94–2.87)	0.08	0.77	0.65
Find and bury bodies	2.12 (1.07–4.20)	0.03	2.20 (1.10–4.37)	0.03	0.64	0.76
Forced to betray family or friend, risking their lives	4.70 (1.08–2.46)	0.04	4.46 (1.04–19.06)	0.04	2.12	0.03
Violent death of spouse or child	1.82 (0.95–3.51)	0.07	1.73 (0.89–3.36)	0.10	0.80	0.61
Death of family/friend	2.12 (1.15–3.90)	0.02	2.15 (1.18–3.94)	0.01	0.74	0.68
Disappearance of spouse/child	1.94 (1.12–3.38)	0.02	1.90 (1.09–3.31)	0.02	2.49	0.008
Disappearance, kidnapping of family/friend	2.44 (1.37–4.33)	0.00	2.72 (1.54–4.82)	0.00	2.75	0.004
Witnessed physical injury of family/friend in combat	1.92 (1.10–3.34)	0.02	1.99 (1.14–3.47)	0.02	2.03	0.03
Witnessed beating/torture of other people	1.83 (0.99–3.38)	0.06	1.82 (0.98–3.38)	0.06	1.52	0.14
Witnessed killing or murder of other people	3.06 (1.73–5.39)	< 0.001	3.28 (1.86–5.79)	< 0.00	0.48	0.89
< =10 traumatic experiences	Ref.					
Between 11 and 15 traumatic experiences	1.20 (1.12–1.29)	< 0.001	1.21 (1.12–1.29)	< 0.001	0.77	0.64
> 15 traumatic experiences	2.11 (1.39–3.19)	< 0.001	2.15 (1.42–3.24)	< 0.001	0.81	0.61

^a^Models were adjusted for age, social support and education level

In adjusted models, depression was significantly associated with several traumatic experiences including rape (OR = 2.3, *p* = 0.01), and experiencing the disappearance/kidnapping of a child or spouse (OR = 1.99, *p* = 0.01) (Table 5).

**Table 5:** Association between depression and traumatic experiences

Type of violence	Depression				Goodness of fit	
	Unadjusted OR (95% CI)	***p***-value	Adjusted OR (95% CI)*	***p***-value	F-statistic	***p***-value
**Sexual violence**						
Rape	2.41 (1.28–4.53)	0.01	2.30 (1.21–4.38)	0.01	1.57	0.12
Gang rape	1.56 (0.75–3.25)	0.24	1.62 (0.78–3.36)	0.20		
Pregnancy resulting from rape	1.96 (1.05–3.68)	0.04	1.70 (0.89–3.23)	0.11	0.85	0.57
Rape before displacement	1.28 (0.71–2.27)	0.41	1.21 (0.67–2.18)	0.52	1.89	0.05
Rape during flight	0.90 (0.50–1.63)	0.73	1.03 (0.57–1.88)	0.91	1.96	0.04
Rape after displacement	1.44 (0.74–2.80)	0.28	1.45 (0.74–2.81)	0.27	1.80	0.07
**Non-sexual traumatic experiences**						
Lack of access to health care	1.71 (0.96–3.07)	0.07	1.71 (0.94–3.11)	0.08	1.58	0.12
Destruction of property	2.59 (1.27–5.31)	0.01	1.88 (0.90–3.94)	0.09	1.74	0.08
Forced labor	1.58 (0.95–2.62)	0.08	1.72 (1.01–2.94)	0.05	0.85	0.57
Forced to betray family or friend, risking their lives	2.30 (0.91–5.82)	0.08	2.43 (0.98–5.99)	0.05	1.96	0.04
Death of family/friend	1.59 (0.96–2.64)	0.07	1.64 (0.98–2.76)	0.06	1.04	0.41
Disappearance, kidnapping of spouse /child	2.13 (1.28–3.53)	< 0.01	1.99 (1.16–3.40)	0.01	2.25	0.02
< =10 traumatic experiences	Ref.					
Between 11 and 15 traumatic experiences	1.25 (0.91–1.73)	0.17	1.19 (0.84–1.67)	0.32	1.19	0.30
> 15 traumatic experiences	1.21 (0.73–1.99)	0.47	1.2- (0.71–2.02)	0.49	1.55	0.13

## Discussion

This is one of the few studies, to our knowledge, utilizing ACASI methodology to describe mental health outcomes among Congolese female refugees living in an urban environment. Among those interviewed, we found that women reported high rates of rape, and that this was associated with higher levels of PTSD and depression. Results highlight important mental and physical health needs of refugee women who, under their host country’s refugee assistance policies, are largely excluded from direct assistance, whether in terms of access to health care or psychosocial support.

The finding that almost 80% of women had experienced rape and that, of those who reported rape, more than half (54%) had experienced more than one incident of rape, underscores the serious threat that women refugees face. The fact that 20% of respondents reported being raped in the country of asylum and that higher rates of PTSD were associated with being raped post-displacements points to serious gaps in the protection afforded to self-settled refugees. Rates of sexual violence in this sample were higher than in similar studies; among adult women fleeing conflict in Liberia (60%), among women living in DRC (16%) [16], and in a previous study of Congolese refugees living in Kampala [10]. The discrepancy in prevalence rates may be the result of several factors, including different populations assessed or differences in the survey tools used. For example, we found higher rates of sexual violence than those reported by Morof and colleagues (2014), who also used RDS methodology to sample hard-to-reach women refugees living in Kampala. This difference could be the result of combining RDS with survey techniques that enable participants to privately answer sensitive questions (ACASI). As in other studies, rape was most frequently perpetrated at the hands of armed groups and often combined with additional traumatic experiences such as destruction of property, forced labor, death of family and kidnappings [17].

Although rape may have been a driver of displacement (60% reported being raped before displacement), more than one third (35%) of women reported being raped in transit, and 20% in the host country. Local policy and services directed to refugees need to recognize that the current practice of providing support primarily to settlement-based refugees in rural areas fails to recognize the multiple needs and safety concerns of women refugees living in urban areas.

Depending on the population sampled, between 30 and 70% of refugees screen positive for PTSD [5, 18], with variations mostly accounted for by population characteristics and survey tools used. Rate of PTSD (73%) based on symptom criteria in this study was higher than that of Congolese women in other studies [7, 10, 19], suggesting the higher need to understand and respond to mental health needs of self-settled refugees. PTSD symptom severity among refugees has been linked to poor executive functioning [9], possibly compromising their psycho-social functioning and ability to navigate complex post-migration environments. As in previous reports among women refugees [10, 20], likelihood of PTSD and depression was greater for women reporting rape and other war-related exposures years after their initial arrival to the host country, even after adjusting for age and sociodemographic factors. The potential for long-lasting symptoms after sexual violence and rape has been described in the Rape Trauma Syndrome (RTS) [21], a nomenclature used to describe post-traumatic stress symptoms that arise from sexual assault not usually diagnosed in PTSD, including sexual dysfunction, long-term depression, fear, anxiety, social maladjustment, and humiliation [22].

Importantly, we found that odds of PTSD were also high among women reporting non-violent experiences such as lacking shelter (OR = 2.86, *p* < 0.01), lacking food or water (OR = 2.53, *p* = 0.02), and lacking access to health care (OR = 2.84, *p* < 0.01). Even in cases where refugees are granted basic rights, their integration prospects are often limited and the effects of sexual and other types of violence on psychological distress are often compounded by the accumulation of post-migration stressors [23] and cultural adaptation [24]. In Burundi and Uganda, for example, DRC refugees have the legal right to work but employment rates are extremely low, limiting their access to livelihoods [25]. By identifying and evaluating post-migration stressors, services and programs in host countries can more effectively manage the critical psychosocial and general health needs of this population, facilitating positive adaptation within host communities.

Given the confined nature and restrictions associated with living in settlements, refugees around the world are increasingly opting to ‘self-settle’ in border areas of large urban hubs [2]. But the extent to which refugees can be agents of development is limited by local government policy allowing their integration. At the time of the survey in 2013, there were an estimated 48,000 Congolese living in Kampala [26], with the majority being recognized by the host Government on a prima facie basis [1]. If provided with appropriate supportive services, including legal, healthcare and psychosocial support, and within a framework of local integration, urban settlements may prove to be better alternatives to camps and succeed where other models have failed [27]. In order to inform local settlement policy, more research with appropriate methodological approaches is needed to understand the current situation and livelihoods of self-settled refugees. For example, qualitative interviews with self-settled Congolese refugees highlight the need for research on the role of social functioning to expand our understanding of social support seeking behaviors from family and community resources that can be harnessed as part of psychosocial interventions [28]. Finally, host countries should support international legal frameworks permitting the safe reporting and prosecution of systematic sexual violence, with sexual violence survivor programs and mental health services included as part of these efforts.

This paper should be viewed in light of a few limitations. We relied on self-reported measures that could introduce social-desirability bias. The survey included sensitive questions and it is possible that participants under-reported traumatic events, including rape. However, the use of ACASI might have helped diminish this as has been the case in other studies [29]. PTSD and depression were classified based on symptom criteria and diagnostic interviews were not conducted. Questions could have been misinterpreted or instruments may not match local constructs of disease. We also lacked measures to examine the access to and uptake of mental health services for this afflicted population in Kampala. Data were collected in 2013 and refugees’ situation and needs may have changed since. However, many Congolese refugees remain in Kampala and given the large influx of additional Congolese refugees to Uganda in 2018 and ongoing [2], we anticipate that the need for psychosocial support given past exposure to trauma as shown here is very likely to remain.

Despite limitations, this paper has several significant strengths. First, this study provides data on mental health outcomes, traumatic experiences and rape among a population of women less represented in other research approaches. Many refugees prefer not to live in camps if they have the option and settle in urban environments but are less represented in the literature. Second, our sample was large and the use of RDS allowed us to generate population-based estimates among a hard to sample group. Third, ACASI interviewing allowed participants to privately answer sensitive and personal questions on a computer with headphones, increasing the likelihood of disclosing traumatic events, including rape.

## Conclusions

In summary, our survey among DR Congolese female refugees self-settled in Kampala sheds light on the shocking frequency of violence, sexual and otherwise, that female refugees endure before, during and after flight, and the high rates of adverse mental health outcomes associated with it. Future programming addressing self-settled refugees should be designed to include services responding to high levels of rape and associated PTSD, as well as ongoing vulnerability to rape in the country of asylum, if they are to succeed.

## Data Availability

The datasets generated and/or analyzed during the current study are not publicly available for ethical reasons but are available from the corresponding author upon reasonable request.

## Abbreviations

ACASI: Audio computer-assisted self-interview
CDC: Centers for Disease Control and Prevention
CI: Confidence Interval
DRC: Democratic Republic of Congo
HTQ: Harvard Trauma Questionnaire
HIV: Human Immunodeficiency Virus
MSPSS: Multidimensional Scale of Perceived Social Support
NGO: Non-governmental organizations
OR: Odds Ratio
PHQ: Patient Health Questionnaire
PTSD: Post-traumatic stress disorder
RDS: Respondent-driven sampling
UNHCR: UN High Commissioner for Refugees
